# The Effect of Drugs on the Intestinal Microbiota in Crohn’s Disease

**DOI:** 10.3390/biomedicines12102241

**Published:** 2024-10-01

**Authors:** Xue Yang, Yinghui Zhang, Caiping Gao, Yan Pan, Shan Du, Shiyu Xiao, Zhou Zhou

**Affiliations:** Department of Gastroenterology, Sichuan Provincial People’s Hospital, School of Medicine, University of Electronic Science and Technology of China, Chengdu 610054, China; yangxue_2001@163.com (X.Y.); zhangyinghui530@163.com (Y.Z.); gaocaip@126.com (C.G.); panyan_08@163.com (Y.P.); constantship@163.com (S.D.)

**Keywords:** Crohn’s disease, mesalazine, azathioprine, infliximab, fecal microbiota, metagenomic analysis

## Abstract

Objective: We took advantage of a single-center cross-sectional study to investigate the effect of different drugs on intestinal microbiota and function in Crohn’s disease. Methods: We studied the difference in fecal microbiota of Crohn’s disease patients treated with mesalazine, azathioprine, and infliximab, as well as untreated patients, by metagenome and screened for differential microbiota. Further, we investigated functional differences in intestinal microbiota among the four groups. Results: Through metagenomic sequencing, we found that there was no difference between the four groups in ACE and Chao1 indices, but IFX and mesalazine improved species diversity and homogeneity compared to the untreated group, as evidenced by statistically significant differences in the Shannon index, Simpson index, and pielou_evenness. In addition, beta diversity suggested a difference between groups, but the difference was not significant. Non-parametric tests revealed differences between the four groups at the phylum level, class level, and genus level. Further analysis by LEfSe analysis revealed that the level of short-chain fatty acid-producing microbiota was increased in the treated groups, while there was no difference between the treated groups when compared to each other. Finally, the KEGG database and EggNOG database revealed that there were functional differences in intestinal microbiota among the four groups, including microbial metabolism pathway, cysteine and methionine metabolism pathway, cytoskeleton, etc. Conclusions: Mesalazine, azathioprine, and infliximab can all affect the intestinal microbiota and function in patients with Crohn’s disease, and the drugs may alleviate intestinal inflammation in patients with Crohn’s disease by modulating the intestinal microbiota.

## 1. Introduction

Crohn’s disease (CD) is a chronic, recurrent intestinal inflammatory disease with increasing incidence in the clinic; repeated inflammation of the intestinal tract can further lead to intestinal mucosal injury, bleeding, perforation and other symptoms, which seriously affects the quality of life of patients [1]. Recent research showed that the development of CD was related to the interactions of intestinal microecology, intestinal epithelial barrier, and immune function [2].

In terms of treatment, mesalazine, azathioprine, hormones, methotrexate, and biologics, such as infliximab, vedolizumab, ustekinumab, and small molecules, are commonly used for the treatment of CD. However, little research has been done on whether the use of drugs affects the intestinal microbiota, and whether there are differences in the effects of different drugs on the intestinal microbiota.

Although there are many drugs that have been developed and are under development, there is no drug that can cure CD. At the same time, each drug has its own advantages and limitations, and the treatment of CD in the future may be more inclined to individual precision therapy, which requires us to have a deeper understanding of the effects of drugs on the disease, on the internal environment of the organism, and especially on the intestinal microbiota. Based on this, our study investigated the effects of different drugs on intestinal microbiota through metagenomic sequencing, which lays the foundation for exploring the mechanism of drug action.

## 2. Materials and Methods

### 2.1. Study Population and Sample Collection

Sixty patients with CD were recruited from the Department of Gastroenterology, Sichuan Provincial People’s Hospital, between April 2020 and November 2023. Inclusion criteria were (1) ileal, colonic, or ileocolonic CD diagnosed by clinical, laboratory, radiological, endoscopic, and histological evidence; (2) no antibiotics, probiotics, prebiotics, etc., received within the previous 4 weeks. Exclusion criteria were (1) pregnancy or lactation status; (2) combination of other malignant tumors. To categorise the location and behaviour of the disease, the Montreal classification was used. All patients provided their written informed consent to participate in this study. After the patients were enrolled, we collected fecal samples in a sterile cup, froze it at −80 °C, and then sent it to a third-party institution for metagenomic sequencing.

### 2.2. Metagenomic Sequencing

Total genomic DNA was extracted from fecal samples using the PowerSoil^®^ DNA Isolation kit (Mo Bio Laboratories, Carlsbad, CA, USA) according to manufacturer’s instructions. After the genomic DNA of the samples was qualified, the DNA was interrupted, and then the fragmented DNA was subjected to product purification, library amplification, and product purification to form a sequencing library, and the library was sequenced by Illumina sequencer after passing quality control. The original reads obtained from sequencing were quality controlled and filtered to obtain clean reads, which were spliced and assembled, coding genes were predicted, non-redundant gene sets were constructed, the non-redundant gene sets were functionally annotated and taxonomically analyzed in both general and specialized databases, and the species composition and abundance information of the samples were collected.

Representative sequences of non-redundant gene catalog were aligned to NCBI NR database with e-value cutoff of 1 × 10^−5^ using Diamond software (version 0.9.29) for taxonomic annotations. KEGG annotation was conducted using Diamond (version 0.9.29) against the Kyoto Encyclopedia of Genes and Genomes database with an e-value cutoff of 1 × 10^−5^. EggNOG annotation was conducted using Diamond software (version 0.9.24) with an e-value cutoff of 1 × 10^−5^.

### 2.3. Statistical Analyses

All statistical analyses were performed using SPSS version 21.0 (IBM Corporation, Armonk, NY, USA). Quantitative data were reported as mean ± standard deviation and qualitative variables were expressed as numbers or percentages. Statistical analyses were performed using the two-tailed non-parametric Mann–Whitney test or Wilcoxon rank-sum test. The linear discriminant analysis effect size (LEfSe) pipeline was used to search for biomarkers with statistical differences. All tests for significance were 2-sided, and differences with *p* < 0.05 were considered statistically significant.

## 3. Results

### 3.1. Characteristics of the Study Population and Fecal Metagenomic Sequencing

Sixty patients with an established diagnosis were recruited in the study between April 2020 and November 2023, and fecal samples were obtained from 54 patients. One sample was excluded due to unqualified metagenomic sequencing data, seven samples were excluded due to less than five patients enrolled in the treatment modality (the number of cases less than five could not be statistically analyzed, include three cases of Ustekinumab and four cases of Vedolizumab). As a result, 46 fecal samples were included in the study, including 13 cases of infliximab (IFX), 7 cases of azathioprine (AZA), 9 cases of mesalazine (M), and 17 cases of untreated (Pre). Their demographic and clinical characteristics are detailed in Table 1. We found no statistically significant differences between the four groups in terms of age, gender, Montreal classification, and perianal lesions. There was no significant statistical difference in the inflammatory indicators of patients, such as ESR and CRP, but there were significant differences in CDAI among the groups. The untreated group was significantly higher than the treated group, indicating that the untreated patients had a higher overall inflammatory activity.

### 3.2. Alpha Diversity and Beta Diversity Analysis between the Groups

We used several methods for alpha diversity assessment of the groups, including ACE index, Chao1 index, Shannon index, Simpson index and pielou_evenness, where the Chao1 index and ACE index were used to measure species richness, Shannon and Simpson indices were used to measure species diversity, and the pielou_evenness index assessed species evenness.

**Figure 1:**
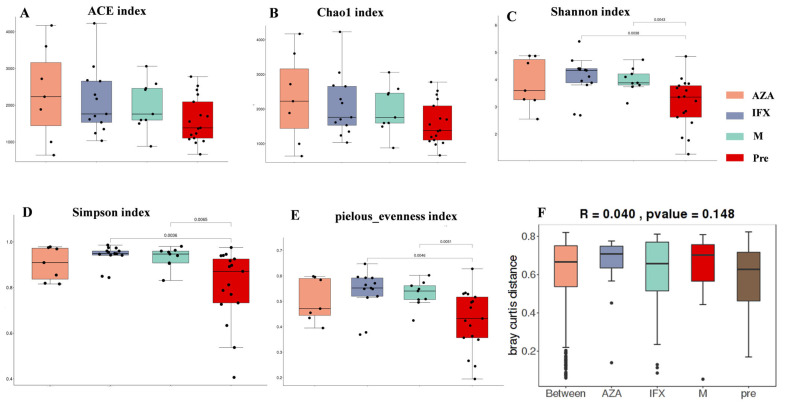
Alpha diversity and beta diversity analysis of fecal microbiota in the four groups. Alpha diversity indices were estimated using the number of observed ACE (**A**), Chao index (**B**), Shannon index (**C**), Simpson index (**D**), and pielou_evenness index (**E**). Beta diversity analysis of fecal microbiota in patients of the four groups (**F**). Significance was determined by the Mann–Whitney U test.

Our study found there were no difference in the Chao1 index and ACE index among the four groups, suggesting that there was no significant difference in species richness (Figure 1A,B). The IFX and M groups were statistically different from the Pre group in Shannon index, Simpson index, and evenness indices (Figure 1C–E), suggesting that infliximab and mesalazine may increase the diversity and homogeneity of intestinal microbiota. Beta diversity analysis was calculated by non-parametric statistical tests. We found difference between groups, but the difference was not significant (R = 0.04, Figure 1F).

### 3.3. Altered Fecal Microbiota between Different Treatment Groups

**Figure 2:**
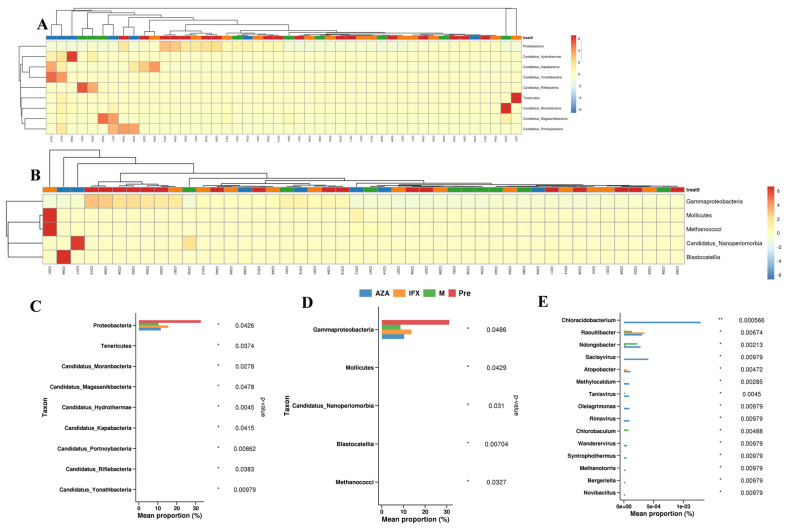
Taxonomic differences in the fecal microbiota in different treatment groups. (**A**) Heatmap of phylum level; (**B**) heatmap of class level; (**C**) bar graph of phylum level; (**D**) bar graph of class level; (**E**) bar graph of genus level. * *p* < 0.05, ** *p* < 0.01.

**Figure 3:**
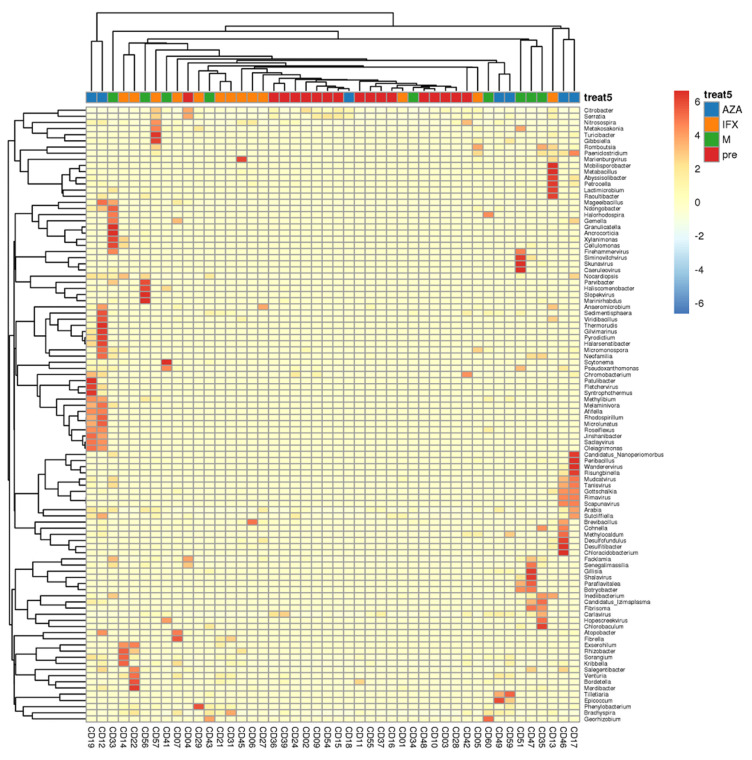
Heatmap of genus level in the fecal microbiota in different treatment groups.

To better understand the changes in fecal microbiota between the four groups, we performed the Mann–Whitney U test comparison, on the order through species levels. Detailed information on all taxonomical levels is shown in Figure 2. We found that the fecal microbiota of the treated groups was significantly different from the Pre group. At the phylum level, we found that the different treatment modalities all resulted in a significant reduction in Proteobacteria phylum levels (Figure 2A,C). At the class level, all three treatment modalities resulted in a decrease in Gammaproteobacteria levels when compared to the Pre group (Figure 2B,D). At the genus level, the three treatments increased the abundance of some species, such as Chloroaceticus, Raoultibacter, Ndongoensis, and also increased the abundance of some viruses, such as Saclayviru, Tanisvirus, Rimavirus, and Wanderervirus (Figure 3, Figure 2E).

### 3.4. LEfSe Analysis of Species between Different Treatment Modalities

**Figure 4:**
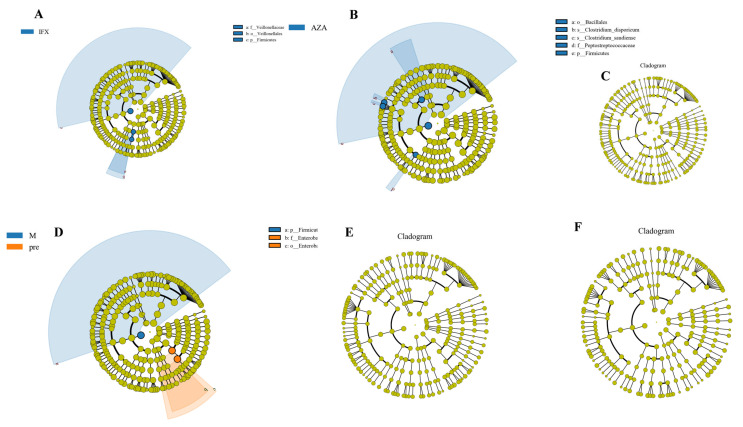
LEfSe analysis of species between different treatment modalities. The circles radiating from the inside to the outside of the evolutionary branching diagram represent the taxonomic levels from kingdom to species; the coloring principle was that the species with no significant differences were uniformly colored in yellow, with significant differences colored in red or blue. (**A**) IFX vs. Pre; (**B**) AZA vs. Pre; (**C**) IFX vs. AZA; (**D**) M vs. Pre; (**E**) IFX vs. M; (**F**) AZA vs. M.

Considering that alterations in gut microbiota from taxa were found in CD patients with different treatments, we further conducted the LEfSe analysis with a significance *p* value of 0.01 to identify the most significantly enriched taxon between the groups. We compared the fecal microbiota of the three treatment modalities with that of Pre group, and found that IFX increased Firmicutes at the phylum level and Veillonellales at the order and family levels (Figure 4A). AZA increased Firmicutes at the phylum level, Bacillales at the order level, Peptostreptococcaceae at the family level, and Clostridium disporicum and Clostridium saudiense at the species level (Figure 4B). Mesalazine increased Firmicutes at the phylum level, whereas Enterobacterales at the order level and Enterobacteriaceae at the family level were more abundant in the Pre group (Figure 4D). In addition, we compared the fecal microbiota of the three treatment groups and found no differences. Therefore, our study found that untreated CD patients have more intestinal pathogenic bacteria, and that different treatments could increase the level of Firmicutes bacteria, Veillonellales, Peptostreptococcaceae, and Clostridium, which are all associated with the production of short-chain fatty acids, suggesting that different treatments might alleviate intestinal inflammation by increasing probiotics and microbiota that improve intestinal metabolism [3,4,5].

### 3.5. Functional Profiling with KEGG Pathway and EggNOG

**Figure 5:**
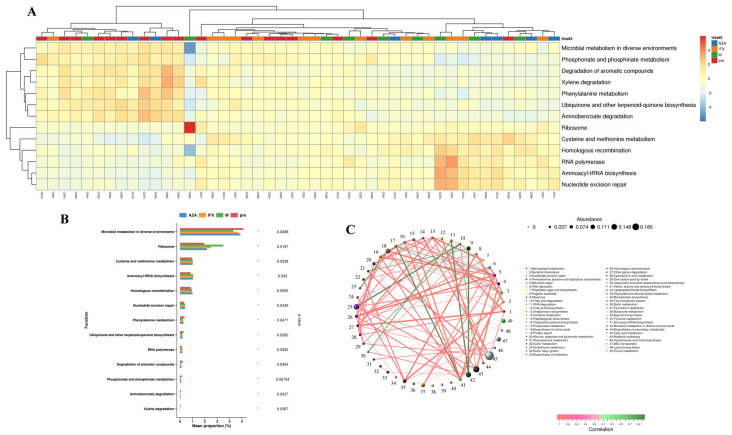
Functional profiling with KEGG pathways. (**A**) Functional genes were tested for non-parametric differences with *p*-values less than 0.05. The right clustering tree is the differential functional gene clustering, the top clustering tree is the sample clustering, and the middle is the heat map. (**B**) Relative abundance of KEGG pathways that were differently distributed in the four group (*p* < 0.05). (**C**) Correlation network diagram is a form of correlation analysis, based on KEGG pathway. * *p* < 0.05.

The Kyoto Encyclopedia of Genes and Genomes (KEGG) database is a database that systematically analyzes the metabolic pathways of gene products in cells and the functions of these gene products [6]. The KEGG database helps to study gene and expression information as a whole network. We used the KEGG database to search for differences in four groups (Figure 5A,B). The main differences in the functional composition of the gut bacteria in the four groups were microbial metabolism (*p* = 0.0288) and cysteine and methionine metabolism (*p* = 0.0328). Previous studies have found that cysteine and methionine metabolism were closely related to intestinal anti-inflammatory functions and maintenance of barrier functions [7,8,9]. This suggested that CD patients might alleviate intestinal inflammation by altering the function of intestinal microbiota.

**Figure 6:**
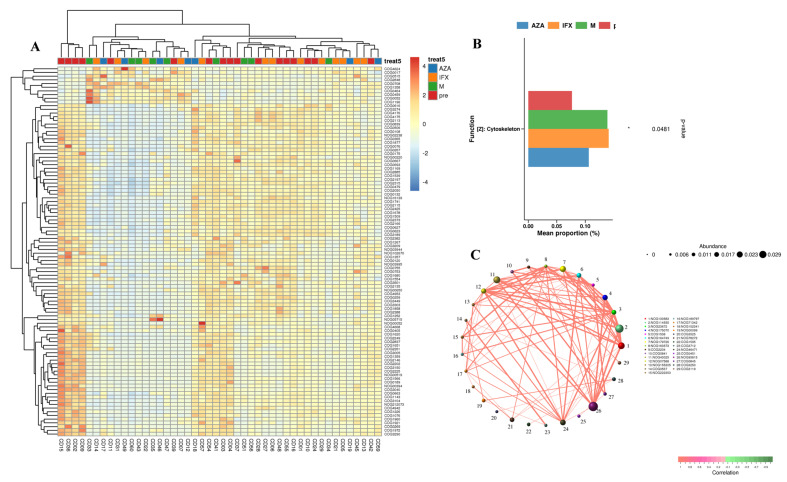
Functional profiling with EggNOG. (**A**) Functional genes were tested for non-parametric differences with *p*-values less than 0.05. The right clustering tree is the differential functional gene clustering, the top clustering tree is the sample clustering, and the middle is the heat map. (**B**) Relative abundance of EggNOG pathways that were differently distributed in the four group (*p* < 0.05). (**C**) Correlation network diagram is a form of correlation analysis, based on EggNOG. * *p* < 0.05.

The Evolutionary Genealogy of Genes: Non-supervised Orthologous Groups (EggNOG) database was used to search for and annotate homologous genes, helping to study the evolution and function of homologous genes [10]. We used the EggNOG database to search for differences in homologous genes between the four groups (Figure 6A,B). The main difference in the functional composition of the gut bacteria of the four groups was in the cytoskeleton, which might be involved in intestinal barrier function, inflammation, and fibrosis through the cytoskeleton [11,12].

Functional gene correlation analysis was based on the pathways of KEGG and the pathways of EggNOG. Through KEGG pathways, we screened the top 49 functional genes with the highest abundance (Figure 5C), based on the abundance and changes of each functional gene in each sample. Through EggNOG pathways, we screened the top 229 functional genes with the highest abundance (Figure 6C), based on the abundance and changes of each functional gene in each sample.

## 4. Discussion

CD is a common chronic, inflammatory, recurrent intestinal disease. Patients mainly manifest symptoms as diarrhea, abdominal pain, bloody stools, and other symptoms, and persons in critical condition can form intestinal perforation, intestinal fistula; this disease occurs repeatedly, affecting the quality life of patients [13]. At this stage, there is no drug that can eradicate CD. In clinical practice, we commonly use medications including mesalazine, azathioprine, biological agents, and so on.

Each drug has a different mechanism of action. Mesalazine exerts its anti-inflammatory and antioxidant effects locally in the colonic mucosa mainly through 5-aminosalicylic acid [14]. Glucocorticoids bind to the glucocorticoid receptor in the cytoplasm and regulate the transcription of anti-inflammatory protein genes, inhibit the activation of pro-inflammatory genes, and induce the degradation of pro-inflammatory gene mRNAs through either nuclear receptor-dependent or non-nuclear receptor-dependent pathways, realizing the anti-inflammatory effect [15]. Azathioprine produces immunosuppressive effects by inhibiting DNA synthesis and thus inhibiting lymphocyte proliferation [16]. As for biological agents, infliximab terminates the biological activity and signaling of anti-tumor necrosis factor by forming a stable complex with human anti-tumor necrosis factor [17]. However, whether these drugs affect the intestinal microbiota, and whether they alleviate intestinal inflammation by modulating the intestinal microbiota, has not been studied.

In this study, we analyzed species richness by alpha and beta diversity. We found that there was no difference between the four groups in ACE and Chao1 indices, but IFX and mesalazine improved species diversity and homogeneity compared to the untreated group, as evidenced by statistically significant differences in the Shannon index, Simpson index, and pielou_evenness. In addition, beta diversity suggested a difference between groups, but the difference was not significant. These results suggested that medication might improve the diversity and balance of the intestinal microbiota.

Then, we searched for differences in fecal microbiota among the four groups by non-parametric analysis, and found that the differences among them mainly existed at the phylum level, the class level, and the genus level. Among them, Proteobacteria was the most important differential species at the phylum and the class level, and the abundance of Proteobacteria was significantly reduced in the treatment groups. At the genus level, the three treatment groups increased the abundance of some strains and viruses, for example, Chloroaceticus, Raoultibacter, Ndongoensis, and Saclayviru, which has not been reported in previous studies. Previous studies have found that in the intestines of CD patients, the abundance of Proteobacteria was increased, which may be associated with the development of CD [18]. In the present study, we found that all the drug treatments were able to reduce the abundance of Proteobacteria, suggesting that in addition to their own anti-inflammatory function, drugs might alleviate intestinal inflammation by altering the intestinal microbiota, and that Proteobacteria was an important microbiota target for the action of drugs.

We further analyzed the differences in fecal microbiota between the treated and untreated groups by LEfSe analysis, and two-by-two comparisons revealed an increase in short-chain fatty acid-producing bacteria in all three treatment modalities, including Firmicutes, Veillonellales, Peptostreptococcaceae, Clostridium, etc. Short-chain fatty acids, which are produced by intestinal microbiota through fermentation of dietary plant fibers that are naturally indigestible in humans, are a source of energy for the colonic epithelium and have been shown to play a key role in maintaining intestinal barrier function and regulating intestinal immune homeostasis [19]. Comparisons between treatment groups did not reveal differences in intestinal microbiota, suggesting that CD therapeutic agents might alleviate intestinal mucosal inflammation by altering the microbiota and improving intestinal metabolism.

We explored the effects of different drugs on gut microbiota function through the KEGG database and the EggNOG database. KEGG is a database that integrates genomic, chemical, and systemic functional information to correlate gene catalogs from genomes that have been completely sequenced with systemic functions at the cellular, species, and ecosystem levels [6]. The EggNOG database is a database of direct homologous proteins based on public resource databases for grouping comparisons, annotated with functional descriptions and functional classifications of orthologous homologous taxa [10].

In our study, certain changes in KEGG pathways and EggNOG were observed in the fecal microbiota of CD patients with different treatments. As for the KEGG pathways, we found that many metabolism pathways had statistically significant differences in expression levels, such as microbial metabolism in diverse environments, ribosome, cysteine and methionine metabolism, aminoacyl-tRNA biosynthesis, homologous recombination, etc. Previous studies have found that cysteine and methionine metabolism was strongly associated with gut health, and that it could alleviate intestinal inflammation by reducing oxidative stress, improving energy status, and had also been implicated in a number of intestinal cell-signaling pathways [20]. The role of other metabolic alterations in intestinal inflammation has not been reported. Thereas, through the EggNOG database, we only found differences in cytoskeletal protein function among the four groups. Previous studies have found that the cytoskeleton was associated with intestinal barrier function, inflammation, and fibrosis [21], suggesting that drugs might have the potential to ameliorate intestinal mucosal inflammation by modulating cytoskeletal-related functions through intestinal microbiota.

This study has several limitations. First, this was a single-center cross-sectional study with a relatively small sample size and a lack of a validation dataset. Second, all the results were based on feces-associated microbiota, and no data from the mucosa-associated microbiota were available. Third, this study lacked the intestinal microbiota at baseline levels. Fourth, there were differences in patients’ medication regimens. Therefore, subsequent proposed prospective studies may go further to clarify the effects of the drugs on the intestinal microbiota of CD patients.

## 5. Conclusions

There are numerous drugs for the treatment of CD, but there are few studies related to the effects of drugs on intestinal microbiota. In this study, we found that the intestinal microbiota of CD was altered after treatment with mesalazine, azathioprine, and infliximab by metagenomic sequencing, and further analyzed the effects of the drugs on the function of the intestinal microbiota by functional database, suggesting that drugs may alleviate the intestinal inflammation of CD by regulating the intestinal microbiota.

## Figures and Tables

**Table 1:** Baseline characteristics of the CD patients.

Characteristics	IFX	AZA	M	Pre	*p*
number	13	7	9	17	
Age (y)	32.92 ± 13.81	37.00 ± 11.73	33.11 ± 16.49	41.71 ± 18.06	0.331
Male/Female (*n*)	6/7	4/11	3/6	12/5	0.169
Montreal classification					
A1/A2/A3 (*n*)	0/10/3	0/4/3	2/5/2	1/8/8	0.265
B1/B2/B3 (*n*)	6/4/3	3/3/1	6/2/1	6/7/4	0.842
L1/L2/L3 (*n*)	3/7/3	3/1/3	1/3/5	3/5/9	0.391
Perianal lesions	4	1	3	5	0.840
ESR (mm/h)	29.50 (11.25, 48.75)	58.00 (31.00, 90.00)	48.00 (26.50, 108.5)	45.00 (18.50, 81.50)	0.150
CRP (mg/L)	4.64 (1.33, 20.19)	6.18 (1.28, 33.97)	17.53 (4.49, 54.47)	14.97 (5.57, 85.43)	0.258
CDAI	58.4 (28.9, 198.6)	82.4 (69.0, 233.0)	188.9 (90.0, 229.0)	223.0 (152.5, 278.5)	0.026

Age is expressed as mean +/− standard deviation; ESR, CRP, and CDAI are expressed as interquartile range.

## Data Availability

The original contributions presented in the study are included in the article, further inquiries can be directed to the corresponding authors.

## Abbreviations

CD, Crohn’s disease; LEfSe, the linear discriminant analysis effect size; IFX, infliximab; AZA, azathioprine; ESR, erythrocyte sedimentation rate; CRP, C-reaction protein; CDAI, Crohn’s disease activity index; KEGG, The Kyoto Encyclopedia of Genes and Genomes; EggNOG, The Evolutionary Genealogy of Genes: Non-supervised Orthologous Groups.
